# A Case of an Unusually Aggressive Cutaneous Anaplastic Large T-Cell Lymphoma in an HIV Patient Treated with CHOP

**DOI:** 10.1155/2011/805893

**Published:** 2011-12-08

**Authors:** Jorge Hurtado-Cordovi, Louay Hanna, Vladimir Gotlieb, Alan S. Multz, Anastasia Pigal

**Affiliations:** ^1^Division of Hematology and Oncology, Department of Medicine, Nassau University Medical Center, North Shore LIJ Health Care System, 2201 Hempstead Turnpike, East Meadow, NY 11554, USA; ^2^Department of Pathology, Nassau University Medical Center, North Shore LIJ Health Care System, 2201 Hempstead Turnpike, East Meadow, NY 11554, USA

## Abstract

Anaplastic large cell lymphoma (ALCL) is the second most common malignancy of T-cell phenotype. This case report describes an unusual rapidly progressing cutaneous anaplastic large T-cell lymphoma in an HIV patient. Our patient is a twenty-year-old African American male with perinatally acquired HIV who presented with a 2 × 2 centimeter necrotic lesion in the right 1st toe; however, 2-3 weeks later multiple smaller lesions appeared on the anterior aspect of the right foot, ankle, and thigh. Biopsy showed cells strongly positive for CD3 and CD30 and negative for CD56 and the ALK gene product. CT of the chest, abdomen, and pelvis was negative for extracutaneous involvement favoring cutaneous ALCL. Patient was treated with 6 cycles of CHOP (cyclophosphamide, hydroxydaunorubicin, vincristine, and prednisone) chemotherapy and went into complete remission. Due to the aggressive course that this malignancy follows in HIV patients we suggest prompt treatment with systemic therapy.

## 1. Introduction

HIV patients are at a higher risk for opportunistic infections and aggressive malignancies. Prior to the highly active antiretroviral therapy (HAART) era, malignant diseases were responsible for 10% of HIV-related deaths [1]. Since the implementation of HAART therapy, it is estimated that 40% of HIV patients are diagnosed with a neoplasm during the course of their illness [1]. While antiretroviral therapy has considerably decreased the incidence of Kaposi sarcoma, the decrease in lymphoma has not been as profound. Non-Hodgkin lymphoma (NHL) is known to be the most common malignancy that occurs in HIV-infected individuals; it has become an AIDS defining disease, and up to 23% of this population succumbed from this illness [1]. According to the World health Organization (WHO), Diffuse Large B Cell lymphoma accounts for roughly 70% of all lymphomas affecting this population, Burkitt lymphoma approximately 20%, and indolent B cell lymphoma, plasmablastic lymphoma, and T cell lymphoma account for the rest; the latest makes less than 3%. Although B-cell NHL is by far the most encountered phenotype, HIV patients are also affected by T-cell malignancies. Linkage of AIDS and cancer registries in the United States has indicated a 15-fold increase in these lymphomas among AIDS patients when compared with the expected incidence in the general population [2]. Anaplastic large cell lymphoma (ALCL) is the second most common type of neoplasm of T-cell origin. It usually presents as primary systemic or cutaneous variant; although morphologically identical, their clinical features and treatments differ. Many experts believe that these two entities are different spectrum of the same disease [2–7].

**Figure 1:**
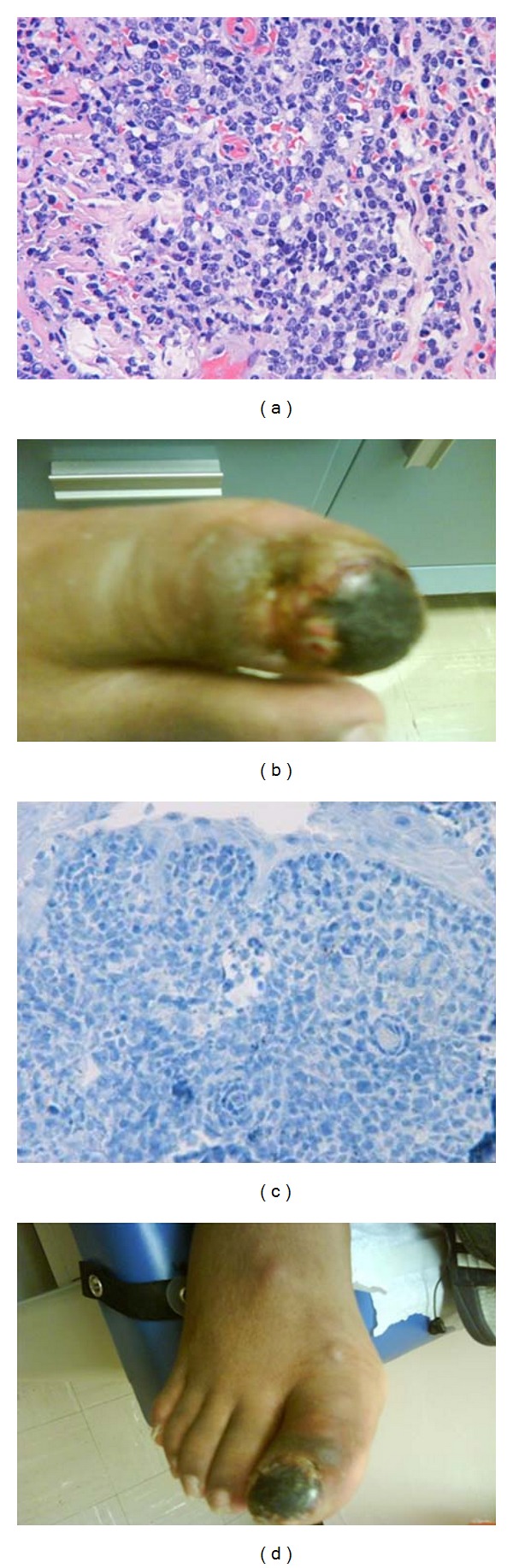
(a) Skin with dense lymphoid infiltrate, consisting of medium to large lymphocytes with dense chromatin, irregular nuclear contours, and occasional prominent nucleoli, shave biopsy (H&E, Original Magnification x400). (b) Patient at presentation with a single necrotic lesion on the big toe. (c) Immunostaining negative for NPM-ALK gene product (original magnification x400). (d) Multiple satellite lesions that appeared 3 weeks after initial presentation.

Histologically, cutaneous ALCL presents with dense lymphocytic infiltrates of the skin (Figure 1(a)). These cells classically exhibit an anaplastic, eccentric, pleomorphic-shaped nucleus with a single or several large nucleoli, abundant cytoplasm, and prominent eosinophilic Golgi apparatus. However, there are other less frequent morphological variants such as small malignant cells with clear cytoplasm and irregular nucleus, sarcomatoid, lymphohistiocytic, eosinophil-rich, and neutrophil-rich variants. Both cutaneous and systemic ALCLs are CD30 positive; it has been hypothesized that this tumor marker may promote the development and survival of malignant clones. Translocation t(2; 5) (p23; q35) known as NPM-ALK encodes for a 80 kilo-Dalton (KDa) tyrosine kinase named Anaplastic Lymphoma Kinase or p80. Cutaneous variant is universally negative for this gene product while systemic ALCL is divided into ALK positive or negative [3]. The absent of this chimeric tyrosine kinase along with its exclusive skin trophism and lack of lymph nodes enlargement are key criteria to differentiate cutaneous versus systemic disease. In addition, laboratory abnormalities that include elevated lactate dehydrogenase (LDH), anemia, and/or thrombocytopenia which are seen in primary systemic are never encountered with cutaneous ALCL [2, 5–7].

There is no consensus on a preferred form of treatment for cutaneous ALCL presenting in HIV/AIDS patients; some experts agree that single, small lesions should be treated with radiation, while multiple lesions should be treated with systemic chemotherapy [8]. This case report presents an unusually rapidly progressing cutaneous anaplastic large T-cell lymphoma in an HIV patient.

## 2. Case Report

Our patient is a twenty-year-old African American male with perinatally acquired HIV; he has been treated and followed by infectious disease since birth. Originally, he presented with a single 2 × 2 centimeter necrotic lesion in the right 1st toe (Figure 1(b)). The patient claims that he first noticed the lesion few weeks ago, and that it has been growing rapidly ever since. At the time of diagnosis patient's CD4 count was 128. Shave biopsy was taken and sent for analysis to the National Institute of Health. The results showed cells strongly positive for CD3 and CD30 and negative for CD 20, CD56, and for p80 tyrosine kinase (Figure 1(c)). CT of the chest, abdomen, and pelvis was negative for extra-cutaneous involvement. Based on the clinical presentation, immunostaining results, and imaging study, the patient was diagnosed with primary cutaneous ALCL. Additional lesions appeared on the lateral aspect of the foot (Figure 1(d)), ankle, and thigh 3 weeks after patient first came to our clinic. After careful evaluation of the literature, we decided to treat the patient with six cycles of CHOP (cyclophosphamide, hydroxydaunorubicin, vincristine, and prednisone).

## 3. Results

**Figure 2:**
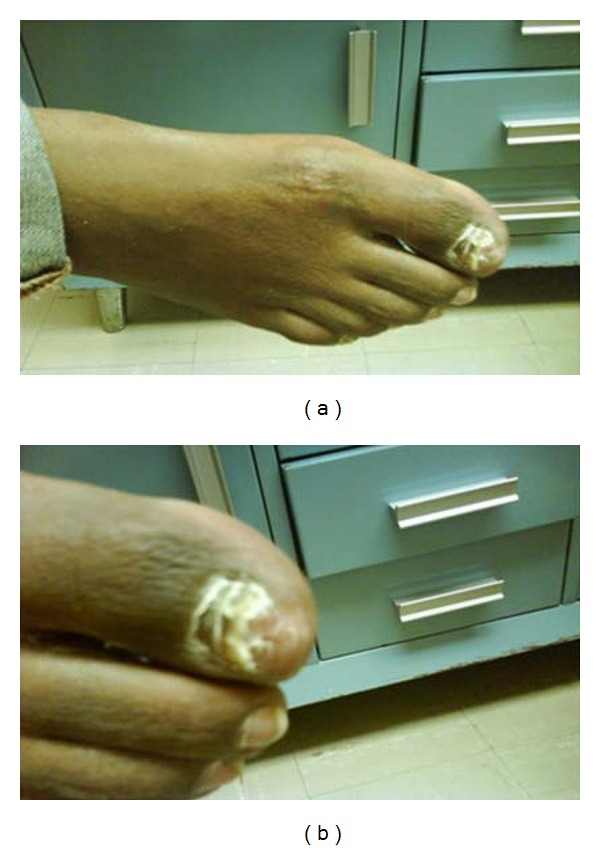
Resolution of all lesions after 6 cycles of CHOP chemotherapy.

Therapy course was complicated by an episode of neutropenia that resolved after a treatment holiday of one week. Each subsequent cycle of chemotherapy was followed by a single injection of pegfilgrastim to prevent treatment delays related to neutropenia. Upon treatment completion, total resolution of the primary lesion located in the first toe as well as secondary lesions as described above was observed (Figures 2(a) and 2(b)).

## 4. Discussion

Non-Hodgkin lymphoma (NHL) has become one of the most common AIDS defining illnesses; although T-cell lymphomas remain rare among HIV patient, they occur more frequently than in immunocompetent individuals [2]. Primary cutaneous ALCL is a rare illness frequently seen in older adults and rarely in pediatric population [5]. It has a tendency to infiltrate the dermis and subcutaneous tissue and typically presents as a solitary, localized skin lesion with or without necrosis or ulceration. However, according to WHO a small percentage of cutaneous ALCL presents with multifocal disease. Malignant infiltration of organs other than the skin is extremely rare; it is occasionally seen in advance untreated cases. Interestingly, this tumor can show spontaneous regression, a behavior commonly seen with single small lesions; unfortunately, it also has a tendency to recur even after complete spontaneous remission [2, 5].

 Neither at the time of diagnosis nor during follow-up did our patient complained of any systemic symptoms, which correlates with the typical indolent presentation of cutaneous ALCL. This seemingly benign onset was contrasted by rapid appearance of multiple smaller satellite lesions with no evidence of systemic disease. It has been hypothesized that defect in cell-mediated immunity and activation secondary to HIV infection is one of the factors responsible for these patient's increased risk for rare, aggressive malignancies. In addition, this viral infection is associated with a cytokine profile change that favors Th2 cytokines production over its Th1 counterpart. The enhanced production of these cytokines increases HIV virus proliferation, virus immune evasion, transcription of some oncogenes, and accumulation of genetic errors favoring the development of premalignant and malignant conditions [9].

In immunocompetent patients, cutaneous variant ALCL commonly presents as solitary, indolent slowly progressing lesion. Radiation therapy is the preferred treatment of solitary or localized cutaneous CD30+ (Ki-1) anaplastic large-cell lymphoma (cutaneous CD30+ ALCL). Doses ranging from 3000 to 3600 cGy are associated with response rates above 90% [9, 10]. Systemic chemotherapy with multiple or single agents is reserved for patients that do not respond to local therapy, for those who have disease presenting as multiple lesions, and for those with extra cutaneous tumor spread [11]. Methotrexate is the most commonly used agent in these settings. Treatment selection for immunocompromised patient can be very challenging. There is very limited literature regarding treatments and their overall effectiveness in HIV patients presenting with multifocal, rapidly progressing disease. However, based on previously published articles by Arzoo et al., systemic chemotherapy alone has been shown to induce complete remission in a few cases of HIV patients affected by this cancer. Based on our patient's clinical presentation and the available research, we decided to treat him with CHOP [6, 11–13].

Unfortunately, this tumor has a tendency to recur, even after long disease-free intervals. According to the literature review CHOP therapy does not prevent future relapses [6, 11]. Newer classes of drugs such as histone deacetylase (HDAC) inhibitors are now available as treatment alternatives. Recently Romidepsin was approved for treatment of cutaneous ALCL for patients that had received systemic chemotherapy at least once [11]. However, the role of this medication is not clear in immunocompromised patients. Immunomodulatory drugs like lenalidomide and thalidomide have been evaluated from the treatment of this malignancy. They inhibit cell growth by inducing cell cycle arrest, reduce the production of proangiogenic cytokines such as vascular endothelial grow factor (VEGF), and stimulate T cells to enhance their antitumor activity. SGN-30 is a chimeric anti-CD 30 monoclonal antibody presently in phase II clinical trials. This novel agent has shown considerable activity against cutaneous ALCL. Bortezomib, a proteasome inhibitor, is active against this malignancy as well. Preclinical data on the use of Bortezomib as single agent chemotherapy for relapsing/refractory T-cell lymphoma have been associated with an overall response rate of 67% [11]. Clinical trials to evaluate the effectiveness of proteasome inhibitors and HDAC combo treatment in relapsing/refractory T-cell NHL, including cutaneous ALCL, are ongoing [6, 11–13]. In some cases autologous and allogeneic bone marrow transplant may be required, but the data are limited [10].

This article describes a case of aggressive cutaneous ALCL in an HIV patient, its management, and clinical outcome. To the best of our knowledge, there are no reported cases of patients with perinatally acquired HIV affected with this illness. The malignant course followed by this neoplasm is also unorthodox. Thus, based on the disease's presentation in our patient and complete remission observed following treatment, we conclude that HIV patients affected by cutaneous ALCL are readily treated with CHOP chemotherapy due to the aggressive course that this tumor tends to follow in this group of patients.
